# Direct Bilirubin Levels and Risk of Metabolic Syndrome in Healthy Chinese Men

**DOI:** 10.1155/2017/9621615

**Published:** 2017-12-20

**Authors:** Xiao-Hong Li, Hai-Yan Lin, Li-Ying Guan, Hui Peng, Meng-Meng Wen, Yong-Qian Cao, Xiu-Yun Jiang, Yi-Bing Wang

**Affiliations:** ^1^Health Management Center, Shandong Provincial Hospital Affiliated to Shandong University, Jinan, Shandong 250021, China; ^2^Department of Burn and Plastic Surgery, Shandong Provincial Hospital Affiliated to Shandong University, Jinan, Shandong 250021, China; ^3^Department of Endocrinology, Shandong Provincial Hospital Affiliated to Shandong University, Jinan, Shandong 250021, China

## Abstract

**Background:**

Serum bilirubin is a potent endogenous antioxidant with anti-inflammatory properties. Several cross-sectional studies have reported that bilirubin was negatively associated with metabolic syndrome. However, in recent longitudinal studies, the relations between bilirubin and metabolic syndrome are inconsistent. Moreover, previous studies mainly focused on serum total bilirubin which is the sum of direct bilirubin and indirect bilirubin. For these reasons, the longitudinal effect of bilirubin subtypes on incident metabolic syndrome was evaluated in Chinese men.

**Methods:**

The study cohort involved 1339 Chinese men without metabolic syndrome. Metabolic syndrome was defined by the American Heart Association/National Heart, Lung and Blood Institute (AHA/NHLBI) criteria, using BMI for the replacement of waist circumference.

**Results:**

There are 117 incident metabolic syndrome cases (8.7%) during 5 years of follow-up among 1339 metabolic syndrome-free participants at baseline. After adjusting for age, drinking, smoking, physical activity, TG, and LDL-C, the odd ratios (ORs) and 95% confidence intervals (CIs) for MetS incidence in the second, third, and fourth quartiles versus the first quartile of DBil concentration were 1.00 (0.61–1.63), 0.57 (0.32–1.02), and 0.51 (0.28–0.92) (*P*_trend_ = 0.031), respectively.

**Conclusions:**

Our findings support the negative association between direct bilirubin and incident metabolic syndrome in healthy Chinese men over 5-year period.

## 1. Introduction

Metabolic syndrome (MetS) is a clustering of metabolic abnormalities characterized by obesity, hypertension, dyslipidemia, and glucose intolerance that appear to increase the risk of diabetes, cardiovascular disease, and overall mortality [1, 2]. Chronic inflammation, oxidative stress, and insulin resistance have been implicated in the underlying pathogenesis [3, 4].

Bilirubin is a product of heme metabolism that may have potent antioxidative property by suppressing oxidization of lipids and lipoprotein [5, 6]. It has also been reported that serum bilirubin exerts anti-inflammatory properties [7]. In line with these findings, elevated serum bilirubin has been found to be negatively related to the oxidative stress and chronic inflammation-related disease such as cardiovascular disease (CVD) [8] and MetS [9–12]. Although the previous studies with cross-sectional design showed a negative association between serum bilirubin and MetS [11], the relation between bilirubin and development of MetS is inconsistent in the longitudinal studies [13, 14]. For example, the report in 2014 by Lee et al. based on a study of 6205 initially health Korean men reported that serum total bilirubin level was negatively associated with incidence of MetS [14]. However, another report of Japanese men and women in 2013 indicated that the total bilirubin is not a risk factor for MetS [13]. Moreover, previous studies mainly focused on serum total bilirubin (TBil) which is the sum of direct bilirubin (DBil) and indirect bilirubin (IBil) [14, 15]. Therefore, it is worthwhile to evaluate the temporal association between TBil, DBil, or IBil and the risk of MetS.

In the present study, we evaluated the prospective association of different circulating forms of bilirubin concentrations, such as total, direct, and indirect, with incident MetS during the 5 years of follow-up period.

## 2. Methods

The study comprised 1804 Chinese men who had undergone routine health examination at Health Management Center of Shandong Provincial Hospital affiliated to Shandong University China in 2011 and who had returned for follow-up examinations in 2016. Among them, 97 subjects with abnormal liver function (defined as a serum aspartate aminotransferase or alanine aminotransferase > 100 U/l, or total bilirubin level > 51.3 *μ*mol/l (3 mg/dl)) [12, 16] or self-reported history of liver disease and cancer were excluded from the analysis. In addition, we further excluded 368 subjects with MetS at baseline. Therefore, 1339 subjects with mean age of 45.6 ± 12.7 years (range: 18–85 years) remained.

Information on age, gender, smoking, alcohol consumption, and history of hypertension and diabetes was obtained from self-reported questions at baseline. Drinking habit was defined by frequency at least once a week. Smoking habit was defined by having ≥5 cigarettes per day. Exercise habit was defined by frequency at least 3 times a week. The physical examination comprised blood pressure (BP) and anthropometric measurements, including height, weight, and BMI. BMI was calculated as weight (kg) divided by height (m)^2^. The venous blood was drawn after 12-hour overnight fasting for examining of fasting plasma glucose (FPG), serum bilirubin (TBil, DBil, and IBil), AST, ALT, r-GGT, and lipids including triglyceride (TG), total cholesterol (CH), high-density lipoprotein cholesterol (HDL-C), and low-density lipoprotein cholesterol (LDL-C). Total white cell counts were performed in the hematology laboratory of our hospital. Serum bilirubin concentration were measured using diazonium salt/diazonium ion with blank method with Beckman Coulter TBil/DBil reagents (Beckman Coulter Test Systems Ltd., Suzhou, China) on Beckman AU5831 autoanalyzer (Beckman Coulter, Inc., California, US). Analytical range of the biochemical autoanalyzer is 0.5–171 *μ*mol/L (0.03–10 mg/dl). Fasting HDL-C was measured by direct method with High-Density Lipoprotein Cholesterol Assay kit (Weihaiwei Biotechnology Ltd., Weihai, China). According to the American Heart Association/National Heart, Lung and Blood Institute (AHA/NHLBI) criteria, using BMI for the replacement of waist circumference, the MetS was defined as having any three or more of the following factors: (a) overweight and/or obesity: BMI ≥ 25.0 kg/m^2^; (b) raised FPG: ≥5.6 mmol/L (100 mg/dL), or being previously diagnosed as type 2 diabetes and taking antiglycemic medication; (c) raised BP: ≥130/85 mmHg, or being previously diagnosed as hypertension and taking antihypertensive medication; (d) raised TG: ≥1.7 mmol/L; (e) reduced HDL-C: <1.03 mmol/L in men [17].

Data analysis was performed by using SPSS 19.0 for Windows. Baseline characteristics data were compared across quartiles of TBil. The distribution of the different variables was examined for normality by the Kolmogorov-Smirnov test. Categorical variables were expressed in percentages and continuous variables in mean (SD) or median (interquartile range). Covariate distributions across baseline TBil quartiles were compared using chi-square test for categorical variables and ANOVA and Kruskal-Wallis test for continuous variables.

Multivariate logistic regression analysis was used to calculate the odds ratios (ORs) and 95% confidence intervals (CI) of incident MetS and individual components of MetS for each bilirubin quartile compared with the lowest quartile, with adjustment for age (continuous), smoking status (yes, no), drinking status (yes, no), and physical activity (yes, no). The CH and LDL-C were treated as continuous variables and included in the final model as previous study [9]. A value of less than 0.05 was deemed statistically significant.

## 3. Results

The median (interquartile range) of serum TBil, DBil, and IBil was 15.41 (15.12, 15.70), 2.75 (2.7, 0 2.81), and 12.66 (12.41, 12.90) *μ*mol/L, respectively. Baseline data according to the quartiles of TBil are presented in Table 1. Participants with high serum TBil concentrations were more likely to be older and nonsmoker and with regular activities. They have low levels of TG and white blood cell count (all *P* < 0.05). However, the TBil concentrations have no significant relationship with the incidence rate of MetS (*P* = 0.134).

A total of 117 MetS cases were identified during the 5 years of follow-up. As show in Table 2, the ORs and 95% confidence intervals (CIs) for incidence MetS in the second, third, and fourth quartiles versus the first quartile of DBil concentration were 1.00 (0.61–1.63), 0.57 (0.32–1.02), and 0.51 (0.28–0.92) (*P*_trend_ = 0.031), respectively. No significant relationship was observed for TBil (*P*_trend_ = 0.066) or IBil (*P*_trend_ = 0.113). The ORs are adjusted for age, drinking, smoking, physical activity, TG, and LDL-C in the final model.

Previous studies have suggested an association between cigarette smoking and low serum bilirubin [18–20]. Therefore, the separate analysis by smoking status was performed. The results showed that DBil concentrations were significantly associated with incident of MetS in either nonsmoking (Table S1) or smoking (Table S2) subjects.

Table 3 shows the adjusted ORs for individual components of MetS according to baseline bilirubin quartile groups during 5-year study period. The incidence of hypertriglyceridemia was negatively correlated with TBil, DBil, and IBil, while the incident of low HDL was negatively correlated with DBil only. The ORs (95% CIs) for incidence hypertriglyceridemia in the second, third, and the fourth quartiles versus the first quartile of TBil, DBil, and IBil concentrations were 0.78 (0.54–1.14), 0.69 (0.47–1.01), 0.57 (0.38–0.84) (*P*_trend_ = 0.033); 0.68 (0.47–0.97), 0.41 (0.28–0.62), 0.48 (0.33–0.42) (*P*_trend_ < 0.0001) and 0.84 (0.58–1.23), 0.81 (0.55–1.22), 0.59 (0.40–0.87) (*P*_trend_ = 0.045), respectively. The ORs (95% CIs) for incidence low HDL in the second, third, and the fourth quartiles versus the first quartile of DBil concentrations were 1.06 (0.68–1.64), 0.85 (0.54–1.34), and 0.53 (0.33–0.87) (*P*_trend_ = 0.018).

## 4. Discussion

In the current study, we found that the DBil levels were associated with a decreased risk of incident MetS. In contrast, no significant associations were found with TBil and IBil levels. To our knowledge, this study was the first perspective study to compare the relationship of MetS development with serum bilirubin (TBil, DBil, and IBil). In addition, serum bilirubin (TBil, DBil, and IBil) was inversely associated with the risk of incident hypertriglyceridemia.

Several cross-sectional and longitudinal studies have reported an inverse association between serum TBil and MetS [10, 11, 14, 21]. For example, a recent cross-sectional study involving 12342 adults in Korean suggested that elevated serum TBil was associated with a decreased risk of MetS [10]. The other 4-year retrospective cohort study in 6205 Korean men indicated that high TBil levels were inversely associated with the development of MetS [14]. In contrast, our study and other Japanese cohort study [13] did not find significant association between TBil and MetS. The bilirubin levels might be the main factor contributing to the inconsistent findings. In the Korean population, more than 50% of the individuals had TBil concentrations higher than 1 mg/dl [14], while, in our present study, 75% of the individuals had TBil concentrations lower than 1 mg/dl (17.1 *μ*mol/L). In the Japanese study which reported no protective association [13], the concentrations of TBil (75% of the individuals had TBil concentrations lower than 1 mg/dl) were similar to our study.

**Figure 1:**
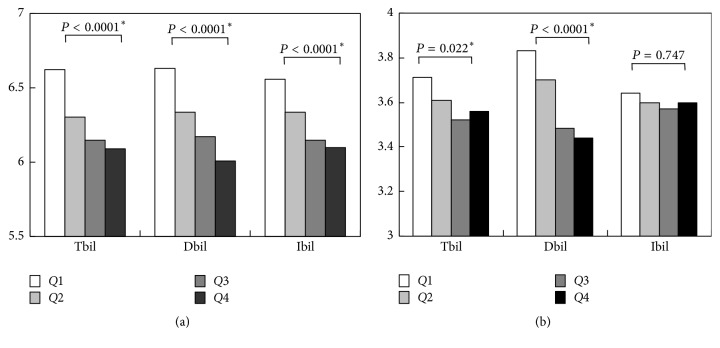
Levels of WBC (a) and LDL-C (b), based on total bilirubin, direct bilirubin, and indirect bilirubin (^*∗*^*P* < 0.05).

In this study, total bilirubin as well as direct and indirect bilirubin was analyzed in relation to MetS. This study indicated that the inverse association of incident MetS was significantly apparent with direct bilirubin. The previous studies have suggested that bilirubin plays a protective role against inflammation and insulin resistance [22]. The inflammatory maker CRP is not routinely measured for heath checking; however, subjects with higher bilirubin including TBil, DBil, and IBil had lower WBC levels in our present study (Figure 1(a)), which is consistent with the previous reports of showing the anti-inflammatory property of bilirubin [23]. Hypertriglyceridemia is well known general aspect of insulin resistance [24]. A significant negative relationship was observed between serum bilirubin and the development of hypertriglyceridemia in our current study. Although serum bilirubin, whether it is total or direct, acts as an indicator of inflammation and insulin resistance in our study, it is unclear why direct bilirubin showed a significant association with the risk of incident MetS.

Recent data have suggested the possible involvement of oxidative stress in the pathogenesis of MetS [25]. Evidence from studies about heme oxygenase (OH) system [26, 27] might support the decreased risk of MetS with elevated direct bilirubin. Bilirubin is produced through the action of heme oxygenase (HO), the rate-limiting enzyme in the catabolism of heme [28]. Formation of bilirubin is inhibited by the downregulation of HO activity [29]. Obesity is associated with insulin resistance and the pathogenesis of T2DM and hypertension and contributes to high levels of LDL-C and triglycerides but low HDL-C levels. In turn, that leads to the development of MetS [30–32]. HDL induces HO-1, and HO-1-mediated decreases in ROS and LDL-C levels were reported in previous diabetes models [26, 33, 34]. Although we did not measure the HO levels and oxidative stress markers in the present population, our results showed that direct bilirubin levels are the most inversely associated with LDL-C concentration (Figure 1(b)). Together with our results showing that DBil concentrations, not total or indirect bilirubin, are negatively correlated with incidence of low HDL, we assumed that direct bilirubin might possess more potential antioxidant properties than the other types of bilirubin. Some studies reported that direct bilirubin is weakly bound to albumin, while indirect bilirubin is strongly bound to albumin [35]; therefore direct bilirubin can be easily separated from albumin and be in active form. Our results show that direct bilirubin concentration was significantly inversely associated with LDL-C levels which might explain the reason why direct bilirubin had a significant inverse relation with MetS. Further studies are needed about the molecular processes underlying effect of direct bilirubin on decreasing the incidence of MetS.

Critically, our results show a positive relationship between serum direct bilirubin and hyperglycemia even though it is not significant. A most recent study found that prediabetes and new-onset diabetes had higher bilirubin levels than subjects with normal fasting glucose, while the bilirubin levels decreased with the prolonged duration of diabetes [36]. Together with the publication showing an inverse association between serum TBil and type 2 diabetes duration [37], it could be explained why our finding is inconsistent with previous cross-sectional study that showed serum bilirubin levels were negatively associated with the development of type 2 diabetes [23, 38, 39].

There are several limitations of the present study. Firstly, the study analyzed subjects who voluntarily visited a health management center and underwent annual follow-up examination; they might not be representative of the general men population. Secondly, instead of waist circumference, BMI was used in the definition of MetS due to a lack of corresponding data. This lack of data could have led to misestimation of the prevalence of MetS. Thirdly, the number of our population was relatively small, and the follow-up periods were relatively short; therefore statistical power might be limited because of the small number of incident cases. Further long-term follow-up prospective studies of a large sample should be conducted to confirm our findings.

In summary, we found that serum direct bilirubin concentrations were negatively associated with the risk of incident MetS in Chinese men. Additional large and long-term prospective studies are required to establish the role of serum bilirubin in the MetS development.

## Figures and Tables

**Table 1:** Baseline characteristics of study subjects based on serum total bilirubin quartile categories.

Variables	Bilirubin quartile catagories				*P* for trend
	*Q*1 (≤11.75 *μ*mol/L)	*Q*2 (11.76–14.30 *μ*mol/L)	*Q*3 (14.31–18.12 *μ*mol/L)	*Q*4 (>18.12 *μ*mol/L)	
	*n* = 335	*n* = 337	*n* = 333	*n* = 334	
Age (years)	43.9 ± 12.3	44.4 ± 12.3	47.0 ± 12.7	47.2 ± 13.2	<0.0001^*∗*^
BMI (kg/m^2^)	25.4 ± 2.6	25.3 ± 2.9	25.3 ± 2.6	24.9 ± 2.7	0.174
Systolic BP (mmHg)	123.6 ± 14.6	123.2 ± 14.1	124.9 ± 15.8	124.2 ± 14.7	0.5
Diastolic BP (mmHg)	73.3 ± 10.5	73.0 ± 9.8	74.9 ± 11.1	73.5 ± 10.3	0.111
Current smoking (%)	35.2	21.6	17.1	18.0	<0.0001^*∗*^
Alcohol drinking (%)	40.8	34.9	39.9	36.2	0.316
Regular exercise (%)	27.5	33.4	37.2	38.6	0.013^*∗*^
FBG (mmol/L)	5.48 ± 0.71	5.51 ± 0.81	5.60 ± 0.91	5.67 ± 1.11	0.039
Total cholesterol (mmol/L)	5.16 (5.04,5.29)	4.97 (4.85,5.09)	5.03 (4.91,5.16)	5.04 (4.92,5.16)	0.06
TG (mmol/L)	1.69 (1.56,1.82)	1.45 (1.33,1.57)	1.41 (1.31,1.51)	1.39 (1.30,1.49)	<0.0001^*∗*^
HDL (mmol/L)	1.20 (1.17,1.24)	1.23 (1.20,1.27)	1.25 (1.21,1.29)	1.28 (1.24,1.31)	0.004^*∗*^
LDL (mmol/L)	3.38 (3.28,3.48)	3.24 (3.15,3.34)	3.28 (3.18,3.38)	3.30 (3.20,3.39)	0.211
ALT (U/L)	27 (25,28)	24 (23,26)	23 (22,25)	24 (23,26)	0.014^*∗*^
AST (U/L)	22 (21,23)	23 (22,23)	22 (22,23)	23 (22,24)	0.568
GGT (U/L)	40 (37,43)	37 (34,40)	34 (31,36)	33 (30,36)	0.002^*∗*^
Total bilirubin (*μ*mol/L)	9.98 (4.04,11.74)	13.10 (11.77,14.32)	16.04 (14.33,18.13)	21.18 (18.15,44.86)	
Direct bilirubin (*μ*mol/L)	1.79 (1.74,1.84)	2.39 (2.35,2.44)	2.89 (2.84,2.94)	3.95 (3.85,4.05)	
Indrect bilirubin (*μ*mol/L)	7.96 (7.82,8.01)	10.07 (10.62,10.77)	13.22 (13.11,13.32)	18.80 (18.37,19.22)	
White blood cell count (10^3^ cells/ml)	6.93 ± 1.44	6.41 ± 1.42	6.29 ± 1.31	6.21 ± 1.32	<0.0001^*∗*^
MetS (%)	9.6	11.0	8.1	6.3	0.134

Data are means ± SD or medians (interquartile range) for skewed variables, or proportions for categorical variables. *P* value was calculated by ANOVA or Kruskal-Wallis test for continuous variables and chi-square test for categorical variables. MetS: metabolic syndrome; BP: blood pressure; BMI: body mass index; FBG: fasting plasma glucose, CH: Total cholesterol; TG: triglyceride; TH: total cholesterol; HDL-C: high-density lipoprotein cholesterol; LDL-C: low-density lipoprotein cholesterol; ALT: aspartate aminotransferase; AST: alanine aminotransferase; GGT: gamma-glutamyltransferase. ^*∗*^*P* < 0.05.

**Table 2:** Associations of serum bilirubin levels and risk of MetS incidence (odds rations and 95% confidence intervals).

	Quartiles of serum bilirubin (mmol/L)				*P* for trend
	*Q*1	*Q*2	*Q*3	*Q*4	
Total bilirubin					
Rang (*μ*mol/L)	≤11.75	11.76–14.30	14.31–18.12	>18.12	
MetS cases/total number of each quartile (%)	32/335	37/337	27/333	21/334	
ORs for MetS					
No adjusted	1	1.20 (0.73–1.98)	0.84 (0.49–1.43)	0.60 (0.34–1.08)	0.09
Model 1	1	1.19 (0.73–1.97)	0.81 (0.47–1.39)	0.58 (0.32–1.03)	0.064
Model 2	1	1.32 (0.79–2.21)	0.87 (0.50–1.52)	0.61 (0.34–1.12)	0.066
Direct bilirubin					
Range	≤2.09	2.10–2.60	2.61–3.22	>3.22	
MetS cases/total number of each quartile (%)	39/335	39/348	20/333	19/333	
ORs for MetS					
No adjusted	1	0.96 (0.60–1.54)	0.50 (0.29–0.88)	0.46 (0.26–0.81)	0.005^*∗*^
Model 1	1	0.95 (0.60–1.53)	0.50 (0.29–0.88)	0.46 (0.26–0.82)	0.006^*∗*^
Model 2	1	1.00 (0.61–1.63)	0.57 (0.32–1.02)	0.51 (0.28–0.92)	0.031^*∗*^
Indirect bilirubin					
Range	≤9.58	9.59–11.76	11.77–14.90	>14.90	
MetS cases/total number of each quartile (%)	32/337	36/334	29/334	20/334	
ORs for MetS					
No adjusted	1	1.15 (0.70–1.90)	0.91 (0.54–1.54)	0.61 (0.34–1.09)	0.145
Model 1	1	1.13 (0.68–1.86)	0.88 (0.52–1.49)	0.58 (0.32–1.03)	0.108
Model 2	1	1.16 (0.69–1.96)	0.92 (0.53–1.58)	0.58 (0.32–1.06)	0.113

Data are expressed as ORs (95% CI). Model 1: adjusted for age; model 2: adjusted further for drinking, smoking, physical activity, CH, and LDL-C. ^*∗*^*P* < 0.05.

**Table 3:** Associations of serum bilirubin levels and risk of MetS individual components incidence (odds rations and 95% confidence intervals).

	Quartiles of serum bilirubin (mmol/L)				*P* for trend
	*Q*1	*Q*2	*Q*3	*Q*4	
Total bilirubin					
Range	≤11.75	11.76–14.30	14.31–18.12	>18.12	
ORs^a^ for each MetS component					
Overweight & obesity	1	0.80 (0.57–1.12)	0.72 (0.51–1.02)	0.76 (0.54–1.08)	0.267
High blood pressure	1	1.18 (0.81–1.74)	1.10 (0.75–1.62)	1.15 (0.79–1.69)	0.833
Hypertriglyceridemia	1	0.78 (0.54–1.14)	0.69 (0.47–1.01)	0.57 (0.38–0.84)	0.033^*∗*^
Low HDL cholesterol	1	1.04 (0.67–1.62)	0.96 (0.61–1.51)	0.84 (0.53–1.33)	0.808
Hyperglycemia	1	1.04 (0.70–1.52)	1.09 (0.74–1.60)	1.08 (0.74–1.58)	0.972
Direct bilirubin					
Range	≤1.90	≤2.09	2.10–2.60	2.61–3.22	
ORs^a^ for each MetS component					
Overweight & obesity	1	0.83 (0.59–1.17)	0.87 (0.61–1.24)	0.81 (0.57–1.15)	0.64
High blood pressure	1	1.08 (0.74–1.56)	1.00 (0.68–1.49)	1.21 (0.82–1.77)	0.746
Hypertriglyceridemia	1	0.68 (0.47–0.97)	0.41 (0.28–0.62)	0.48 (0.33–0.72)	<0.0001^*∗*^
Low HDL cholesterol	1	1.06 (0.68–1.64)	0.85 (0.54–1.34)	0.53 (0.33–0.87)	0.018^*∗*^
Hyperglycemia	1	1.23 (0.84–1.79)	1.06 (0.71–1.59)	1.48 (1.00–2.18)	0.198
Indirect bilirubin					
Range	≤9.58	9.59–11.76	11.77–14.90	>14.90	
ORs^a^ for each MetS component					
Overweight & obesity	1	0.82 (0.59–1.16)	0.81 (0.57–1.14)	0.75 (0.53–1.06)	0.403
High blood pressure	1	1.14 (0.78–1.67)	1.18 (0.81–1.73)	1.14 (0.78–1.66)	0.846
Hypertriglyceridemia	1	0.84 (0.58–1.23)	0.81 (0.55–1.22)	0.59 (0.40–0.87)	0.045^*∗*^
Low HDL cholesterol	1	0.96 (0.62–1.51)	1.01 (0.65–1.58)	0.85 (0.54–1.35)	0.882
Hyperglycemia	1	0.82 (0.55–1.20)	1.04 (0.71–1.53)	0.93 (0.63–1.36)	0.611

Data are expressed as ORs (95% CI). ^a^Adjusted for age, drinking, smoking, physical activity, CH, and LDL-C. ^*∗*^*P* < 0.05.

## Supplementary Materials

Supplementary Materials Separate analysis by smoking status was performed. Association of serum bilirubin levels and risk of MetS incidence has been shown in nonsmoking (Table S1) and smoking subjects (Table S2), separately. Table S1: associations of serum bilirubin levels and risk of MetS incidence in nonsmoking subjects (odds rations and 95% confidence intervals). Table S2: associations of serum bilirubin levels and risk of MetS incidence in smoking subjects (odds rations and 95% confidence intervals).

## References

[1] Laaksonen D. E., Lakka H.-M., Niskanen L. K., Kaplan G. A., Salonen J. T., Lakka T. A. (2002). Metabolic syndrome and development of diabetes mellitus: application and validation of recently suggested definitions of the metabolic syndrome in a prospective cohort study. *American Journal of Epidemiology*.

[2] Isomaa B., Almgren P., Tuomi T. (2001). Cardiovascular morbidity and mortality associated with the metabolic syndrome. *Diabetes Care*.

[3] Houstis N., Rosen E. D., Lander E. S. (2006). Reactive oxygen species have a causal role in multiple forms of insulin resistance. *Nature*.

[4] Lugrin J., Rosenblatt-Velin N., Parapanov R., Liaudet L. (2014). The role of oxidative stress during inflammatory processes. *biological chemistry*.

[5] Stocker R., Yamamoto Y., McDonagh A. F. (1987). Bilirubin is an antioxidant of possible physiological importance. *Science*.

[6] McDonagh A. F. (2010). The biliverdin-bilirubin antioxidant cycle of cellular protection: Missing a wheel?. *Free Radical Biology & Medicine*.

[7] Vítek L. (2012). The role of bilirubin in diabetes, metabolic syndrome, and cardiovascular diseases. *Frontiers in Pharmacology*.

[8] Djoussé L., Levy D., Cupples L. A., Evans J. C., D'Agostino R. B., Ellison R. C. (2001). Total serum bilirubin and risk of cardiovascular disease in the Framingham Offspring Study. *American Journal of Cardiology*.

[9] Lin L.-Y., Kuo H.-K., Hwang J.-J. (2009). Serum bilirubin is inversely associated with insulin resistance and metabolic syndrome among children and adolescents. *Atherosclerosis*.

[10] Choi S. H., Yun K. E., Choi H. J. (2013). Relationships between serum total bilirubin levels and metabolic syndrome in Korean adults. *Nutrition, Metabolism & Cardiovascular Diseases*.

[11] Jo J., Yun J. E., Lee H., Kimm H., Jee S. H. (2011). Total, direct, and indirect serum bilirubin concentrations and metabolic syndrome among the Korean population. *Endocrine Journal*.

[12] Hwang H. J., Kim S. H. (2010). Inverse relationship between fasting direct bilirubin and metabolic syndrome in Korean adults. *Clinica Chimica Acta*.

[13] Oda E., Aizawa Y. (2013). Total bilirubin is inversely associated with metabolic syndrome but not a risk factor for metabolic syndrome in Japanese men and women. *Acta Diabetologica*.

[14] Lee M. J., Jung C. H., Kang Y. M. (2014). Serum bilirubin as a predictor of incident metabolic syndrome: A 4-year retrospective longitudinal study of 6205 initially healthy Korean men. *Diabetes & Metabolism*.

[15] Lee Y.-B., Lee S.-E., Jun J. E. (2016). Change in Serum Bilirubin Level as a Predictor of Incident Metabolic Syndrome. *PLoS ONE*.

[16] Ong K. L., Wu B. J., Cheung B. M. Y., Barter P. J., Rye K.-A. (2011). Association of lower total bilirubin level with statin usage: The United States National Health and Nutrition Examination Survey 1999-2008. *Atherosclerosis*.

[17] Grundy S. M., Cleeman J. I., Daniels S. R. (2005). Diagnosis and management of the metabolic syndrome: an American Heart Association/National Heart, Lung, and Blood Institute scientific statement. *Circulation*.

[18] Schwertner H. A. (1998). Association of smoking and low serum bilirubin antioxidant concentrations. *Atherosclerosis*.

[19] Bulmer A. C., Verkade H. J., Wagner K. H. (2013). Bilirubin and beyond: a review of lipid status in Gilbert's syndrome and its relevance to cardiovascular disease protection. *Progress in Lipid Research*.

[20] Jo J., Kimm H., Yun J. E., Lee K. J., Jee S. H. (2012). Cigarette smoking and serum bilirubin subtypes in healthy Korean men: The Korea Medical Institute Study. *Journal of Preventive Medicine & Public Health*.

[21] Zhong P., Sun D. M., Wu D. H., Li T. M., Liu X. Y., Liu H. Y. (2017). Serum total bilirubin levels are negatively correlated with metabolic syndrome in aged Chinese women: A community-based study. *Brazilian Journal of Medical and Biological Research*.

[22] Jung C. H., Lee M. J., Kang Y. M. (2014). Higher serum bilirubin level as a protective factor for the development of diabetes in healthy Korean men: A 4 year retrospective longitudinal study. *Metabolism - Clinical and Experimental*.

[23] Ohnaka K., Kono S., Inoguchi T. (2010). Inverse associations of serum bilirubin with high sensitivity C-reactive protein, glycated hemoglobin, and prevalence of type 2 diabetes in middle-aged and elderly Japanese men and women. *Diabetes Research and Clinical Practice*.

[24] Grundy S. M. (1999). Hypertriglyceridemia, insulin resistance, and the metabolic syndrome. *American Journal of Cardiology*.

[25] Roberts C. K., Sindhu K. K. (2009). Oxidative stress and metabolic syndrome. *Life Sciences*.

[26] Burgess A., Li M., Vanella L. (2010). Adipocyte heme oxygenase-1 induction attenuates metabolic syndrome in both male and female obese mice. *Hypertension*.

[27] Mölzer C., Wallner M., Kern C. (2017). Characteristics of the heme catabolic pathway in mild unconjugated hyperbilirubinemia and their associations with inflammation and disease prevention. *Scientific Reports*.

[28] Lin J.-P., Vitek L., Schwertner H. A. (2010). Serum bilirubin and genes controlling bilirubin concentrations as biomarkers for cardiovascular disease. *Clinical Chemistry*.

[29] Kappas A. (2004). A Method for Interdicting the Development of Severe Jaundice in Newborns by Inhibiting the Production of Bilirubin. *Pediatrics*.

[30] Fain J. N., Madan A. K., Hiler M. L., Cheema P., Bahouth S. W. (2004). Comparison of the release of adipokines by adipose tissue, adipose tissue matrix, and adipocytes from visceral and subcutaneous abdominal adipose tissues of obese humans. *Endocrinology*.

[31] Tiwari S., Ndisang J. F. (2014). The heme oxygenase system and type-1 diabetes. *Current Pharmaceutical Design*.

[32] Mishra M., Ndisang J. F. (2014). A critical and comprehensive insight on heme oxygenase and related products including carbon monoxide, bilirubin, biliverdin and ferritin in type-1 and type-2 diabetes. *Current Pharmaceutical Design*.

[33] Kruger A. L., Peterson S., Turkseven S. (2005). D-4F induces heme oxygenase-1 and extracellular superoxide dismutase, decreases endothelial cell sloughing, and improves vascular reactivity in rat model of diabetes. *Circulation*.

[34] Cao J., Inoue K., Sodhi K. (2012). High-fat diet exacerbates renal dysfunction in SHR: reversal by induction of HO-1-adiponectin axis. *Obesity*.

[35] Nakagami T., Toyomura K., Kinoshita T., Morisawa S. (1993). A beneficial role of bile pigments as an endogenous tissue protector: anti-complement effects of biliverdin and conjugated bilirubin. *Biochimica et Biophysica Acta*.

[36] Wang J., Li Y., Han X. (2017). Serum bilirubin levels and risk of type 2 diabetes: Results from two independent cohorts in middle-aged and elderly Chinese. *Scientific Reports*.

[37] Chung J. O., Cho D. H., Chung D. J., Chung M. Y. (2015). The duration of diabetes is inversely associated with the physiological serum bilirubin levels in patients with type 2 diabetes. *Internal Medicine*.

[38] Cheriyath P. (2010). High Total Bilirubin as a Protective Factor for Diabetes Mellitus: An Analysis of NHANES Data From 1999 - 2006. *Journal of Clinical Medicine Research*.

[39] Han S. S., Na K. Y., Chae D.-W., Kim Y. S., Kim S., Chin H. J. (2010). High serum bilirubin is associated with the reduced risk of diabetes mellitus and diabetic nephropathy.. *The Tohoku Journal of Experimental Medicine*.

