# Supplementary material for: Direct Bilirubin Levels and Risk of Metabolic Syndrome in Healthy Chinese Men
Source: Biomed Res Int. 2017 Dec 20;2017:9621615. doi: 10.1155/2017/9621615 (PMC5750483; doi:10.1155/2017/9621615)
Supplement: Supplementary Materials — Separate analysis by smoking status was performed. Association of serum bilirubin levels and risk of MetS incidence has been shown in nonsmoking (Table S1) and smoking subjects (Table S2), separately. Table S1: associations of serum bilirubin levels and risk of MetS incidence in nonsmoking subjects (odds rations and 95% confidence intervals). Table S2: associations of serum bilirubin levels and risk of MetS incidence in smoking subjects (odds rations and 95% confidence intervals). [file 9621615.f1.pdf]

Table S1: Associations of serum bilirubin levels and risk of MetS incidence in non-smoking subjects (odds rations and 95% confidence intervals)

		Quartiles of serum bilirubin (mmol/L)				
		Q1	Q2	Q3	Q4	P for trend
Total bilirubin	Range（µmol/L）	≤12.18	12.19 -14.80	14.81-18.40	＞18.41	
	Mets cases/total number of each quartile (%)	26/259	25/259	13/256	17/257	
	ORs for Mets					
	No adjusted	1	0.96（0.54-1.71）	0.48（0.24-0.96）	0.64（0.34-1.20）	0.097
	Model 1	1	0.92（0.51-1.64）	0.45（0.22-0.90）	0.58（0.31-1.11）	0.06
	Model 2	1	1.18（0.63-2.20）	0.54（0.26-1.12）	0.72（0.37-1.43）	0.127
Direct bilirubin	Range	≤2.16	2.17 -2.70	2.71-3.30	＞3.30	
	Mets cases/total number of each quartile (%)	30/259	23/279	16/247	12/246	
	ORs for Mets					
	No adjusted	1	0.69（0.39-1.22）	0.53（0.28-1.00）	0.39（0.20-0.78）	0.035*
	Model 1	1	0.67（0.38-1.20）	0.52（0.28-0.99）	0.40（0.20-0.80）	0.038*
	Model 2	1	0.81（0.44-1.49）	0.65（0.33-1.27）	0.50（0.24-1.05）	0.272
Indirect bilirubin	Range	≤9.90	9.91 -12.10	12.11-15.10	＞15.10	
	Mets cases/total number of each quartile (%)	18/261	27/259	15/255	17/256	
	ORs for Mets					
	No adjusted	1	1.22（0.67-2.28）	0.68（0.34-1.34）	0.77（0.40-1.49）	0.229
	Model 1	1	1.22（0.67-2.21）	0.64（0.32-1.27）	0.70（0.36-1.36）	0.168
	Model 2	1	1.36（0.72-2.58）	0.72（0.35-1.48）	0.78（0.39-1.57）	0.226

Data are expressed as ORs (95% CI). Model 1: adjusted for age; model 2: adjusted further for drinking, smoking, physical activity, CH and LDL-C. *, $P < 0.05$.

Table S2: Associations of serum bilirubin levels and risk of MetS incidence in smoking subjects (odds rations and 95% confidence intervals)

		Quartiles of serum bilirubin (mmol/L)				
		Q1	Q2	Q3	Q4	P for trend
Total bilirubin	Range（μmol/L）	≤10.36	10.37 -13.02	13.03-16.53	＞16.53	
	Mets cases/total number of each quartile (%)	12/77	13/77	7/78	4/76	
	ORs for Mets					
	No adjusted	1	1.10（0.47-2.59）	0.53（0.20-1.44）	0.30（0.09-0.98）	0.067
	Model 1	1	1.13（0.48-2.66）	0.55（0.20-1.49）	0.31（0.10-1.01）	0.074
	Model 2	1	1.10（0.45-2.70）	0.50（0.18-1.42）	0.29（0.08-0.97）	0.066
Direct bilirubin	Range	≤1.80	1.81 -2.30	2.31-3.00	＞3.00	
	Mets cases/total number of each quartile (%)	15/77	11/83	7/72	3/76	
	ORs for Mets					
	No adjusted	1	0.63（0.27-1.48）	0.45（0.17-1.17）	0.17（0.05-0.61）	0.018*
	Model 1	1	0.65（0.28-1.52）	0.46（0.17-1.19）	0.17（0.05-0.62）	0.019*
	Model 2	1	0.74（0.30-1.81）	0.42（0.15-1.16）	0.14（0.04-0.53）	0.008*
Indirect bilirubin	Range	≤8.5	8.51 -10.74	10.75-13.50	＞13.50	
	Mets cases/total number of each quartile (%)	12/78	12/76	878	4/76	
	ORs for Mets					
	No adjusted	1	1.03（0.43-2.46）	0.63（0.24-1.64）	0.31（0.09-1.00）	0.113
	Model 1	1	1.05（0.44-2.51）	0.65（0.25-1.70）	0.31（0.10-1.02）	0.126
	Model 2	1	1.09（0.44-2.72）	0.60（0.22-1.62）	0.29（0.09-0.99）	0.098

Data are expressed as ORs (95% CI). Model 1: adjusted for age; model 2: adjusted further for drinking, smoking, physical activity, CH and LDL-C. *, $P < 0.05$.
